# The role of the nursing work environment, head nurse leadership and presenteeism in job embeddedness among new nurses: a cross-sectional multicentre study

**DOI:** 10.1186/s12912-024-01823-1

**Published:** 2024-03-05

**Authors:** Sisi Fan, Siqi Zhou, Jun Ma, Wenhong An, Honghong Wang, Tao Xiao

**Affiliations:** 1 The Third Xiangya Hospital, Central South University, Changsha, China; 2 Xiangya School of Nursing, Central South University, Changsha, China

**Keywords:** New nurse retention, Nursing work environment, Head nurse leadership, Presenteeism, Job embeddedness, Sequential multiple mediations

## Abstract

**Background:**

The retention of new nurses has become a major challenge for medical institutions. Job embeddedness has been seen as a valuable lens for examining nurse turnover, but greater details about job embeddedness are rarely disclosed, especially among new nurses. This study aimed to reveal how the nursing work environment, head nurse leadership and presenteeism shape job embeddedness in this population from the perspective of conservation of resources (COR) theory.

**Method:**

A cross-sectional multicentre study involving 436 participants from 10 cities and 33 hospitals was conducted over 4 months. Samples were selected using a two-stage convenience sampling method. A sequential multiple mediation model performed with SPSS-PROCESS was used to analyse the relationships among the nursing work environment, head nurse leadership, presenteeism and job embeddedness.

**Results:**

The nursing work environment not only directly affects the job embeddedness of new nurses (*β* = 0.480, *p* < 0.001) but also indirectly affects it through the sequential multiple mediating effects of head nurse leadership and presenteeism (R^2^ = 0.535, F = 82.160, *p* < 0.001).

**Conclusions:**

New nurses’ job embeddedness needs to be improved. These results suggest that preserving adequate resources for new nurses, such as work environment resources, head nurse leadership resources, and individual productivity resources, is an effective way to shape their job embeddedness. In addition, when a certain resource is insufficient, fully considering the principles of investment and buffering between resources and providing reciprocal, alternative, or buffer resources in a timely manner are necessary to improve new nurses’ job embeddedness.

**Large language models:**

Large language models (LLMs), such as ChatGPT, were not used during the writing of this article. An expert native English speaker performed language revision.

**Supplementary Information:**

The online version contains supplementary material available at 10.1186/s12912-024-01823-1.

## Introduction

The retention of new nurses has become a serious challenge worldwide. A systematic review involving 9029 nurses revealed that the annual turnover rate of newly licenced registered nurses ranged from 12 to 25%, which is even greater than that of experienced nurses [1]. In the United States, the average turnover rate of registered nurses in 2021 was 27.1%, with 59% leaving within one year and 74.7% within two years [2]. In China, the turnover rate of new nurses is higher than that of experienced nurses, ranging from 9.4–60% [3, 4]. The departure of new nurses imposes a substantial economic cost, resulting in an average cost to hospitals of more than USD 5 million per year [2]. In addition, training new nurses increases hospital costs, which increases the difficulty of recruiting skilled nurses and ultimately reduces the quality of care [5]. Retaining new nurses is an important aspect of human resources management. Therefore, exploring the potential factors influencing new nurse retention is necessary to overcome the serious problem of new nurse attrition.

Job embeddedness, as a new perspective for assessing nurse retention, predicts and explains turnover better than traditional attitudinal variables such as job satisfaction or organizational commitment because millennials, who represent a predominant generation among current new graduate nurses, are often more engaged than are loyal individuals [6, 7]. Job embeddedness is the closeness of the network of relationships between individuals and all job-related situations inside and outside the organization and indicates the extent to which members are attached to their organizations [8]. There are three key components of this factor: fit, link and sacrifice. Fit refers to the compatibility and comfort of employees with the organization’s environment. Link refers to the formal or informal connection an employee has with others, the community, and other activities, while sacrifice refers to the loss an employee would face by leaving the organization [9]. The role of job embeddedness in nurse career retention has been widely recognized. A meta-analysis reviewing 417 studies revealed that job embeddedness was a predictor of turnover [10] and could explain the voluntary turnover of nurses [11]. Nurses with good job embeddedness tend to have a higher retention rate [12, 13]. Research indicates that nurses’ job embeddedness is shaped primarily during the first two years of employment, as the transition period during which a new nurse becomes a qualified nurse is considered a time of vulnerability. During this period, providing them with resources such as technical guidance, organizational support, and career planning can enhance their sense of belonging and make them better embedded in the organization [14]. Therefore, job embeddedness may be a breakthrough point in solving the problem of the retention of new nurses.

There is limited exploration of new nurses’ job embeddedness. Current research focuses on identifying influencing factors. A review noted that the main factors influencing nurses’ job embeddedness were the work environment, such as corporate culture and staffing; leadership behaviours, such as leadership style and support for subordinates; and personal characteristics, such as working years, psychological characteristics and personal productivity [15]. In addition, one study explored the mechanism of nurses’ job embeddedness and found that servant leadership could shape nurses’ job embeddedness through psychological contract fulfilment and psychological ownership [16]. However, the mechanism underlying the embeddedness of new nurses is unclear. Thus, clarifying the influencing factors and shaping mechanism of new nurses’ job embeddedness can provide ideas for new nurse retention policies.

The conservation of resources (COR) theory aims at employee retention and emphasizes that individuals tend to meet internal and external needs and achieve their goals by acquiring, protecting and retaining resources [17]. Subsequently, Kiazad used the COR theory to explain how people embed their work. They believed that protecting and accumulating resources are key mechanisms for job embeddedness. A high degree of job embeddedness is a resource-rich state in which the link dimension and the fit dimension of job embeddedness represent relational resources and belonging resources, respectively, and the sacrifice dimension represents resource loss [18]. Employees use these tangible and intangible resources to form a network of contacts with the organization [18, 19]. Therefore, this study aimed to use COR theory as a primary theoretical framework to clarify the potential process of shaping job embedding among new nurses.

## Background

According to COR theory, people stay because they are compatible with the work environment, and work environment resources are the basic resources for employee embedding [19]. The nursing work environment refers to a set of characteristics of a work setting that foster or hamper professional nursing practices [20]. A study showed that the foundation of quality nursing, cooperation between nurses and doctors, nurse participation in hospital management, and sufficient manpower and materials are highly correlated with job embeddedness [13]. The overall nursing work environment is positively correlated with the level of nurse job embeddedness [12, 21] since a supportive nursing work environment, such as good staffing, supportive team morale, and promising professional development, provides nurses with purpose and meaning in their work. These factors increase attachment to the organization and lead to the formation of close connections between the nurse and the work relationship network [14]. Therefore, to appeal to their study, we hypothesized that the nursing work environment positively affects the job embeddedness of new nurses.

On the one hand, resource investment, which states that people invest resources to obtain more resources and that the growth of one resource is beneficial to the growth of another resource, is an important principle of COR theory [19]. Numerous studies have shown that a good nursing work environment contributes to the development of head nurses’ leadership [22, 23]. In addition, head nurses’ leadership, as a relational resource, also contributes to nurses’ job embeddedness [16]. For example, service-oriented leaders can develop psychological contracts with nurses, increase their psychological sense of belonging, and cultivate job embeddedness [16]. Humble leadership helps nurses feel respected and enhances their sense of professional value, improving their job embeddedness [24]. In other words, the accumulation of resources in the nursing work environment is beneficial for the acquisition of resources by heads, which ultimately helps nurses become embedded in their work. Therefore, to appeal to the above studies, we hypothesized that head nurse leadership is a mediator of the nursing work environment and the job embeddedness of new nurses.

On the other hand, the primacy of loss is another important principle of COR theory [19]. The motive of loss aversion explains buffered embedding, which means that resources in one domain can be commandeered to buffer resource losses in another domain, ultimately achieving buffered embeddings [18]. Presenteeism refers to the loss of productive resources and is defined as a decrease in productivity and below-normal work quality; although workers are physically present at work, they are mentally absent and unable to participate fully [25]. Presenteeism was found to be more common in new nurses than in those with more experience [26]. One study suggested that nurses with high presenteeism have lower job embeddedness since presenteeism leads to false job engagement and may prevent nurses from fully engaging in their work [27]. Moreover, studies have proposed that the nursing work environment is an influencing factor of presenteeism; that is, a good nursing work environment can reduce presenteeism [28, 29]. Therefore, we speculate that nurses may command a work environment to buffer the loss of productivity and eventually achieve buffered embedding. In response to their research call, we posit that presenteeism is a mediator of the nursing work environment and job embeddedness of new nurses.

Hobfoll suggested that resources do not exist in isolation but can be taught, cultivated and allowed to flow among each other [19]. Further elucidating the relationship between resources is the key to clarifying the path of new nurses’ job embedding. A study of 2,291 nurses demonstrated that nurses’ worse perception of their work environment was related to a lower evaluation of head nurse leadership [22]. An overly authoritative leadership style can lead to more serious presenteeism [30]. Nurses’ presenteeism may prevent them from fully engaging in their work, eventually leading to false job embeddedness [27]. Based on the above studies, we hypothesized that head nurse leadership and presenteeism have sequential mediating effects on the relationship between the nursing work environment and nurse embeddedness.

In summary, based on the perspective of COR theory, this study comprehensively considers the interaction between resources and clarifies the mechanism by which the nursing work environment, head nurse leadership, and absenteeism jointly shape the job embeddedness of new nurses. This study is the first to focus on job embeddedness among new nurses and reveals the shaping mechanism of job embeddedness among new nurses from a theoretical perspective, providing a basis for understanding and intervening in new nurses’ job embeddedness.

### Aims and hypotheses

This study aimed to reveal the underlying mechanisms of the effects of the nursing work environment, head nurse leadership and presenteeism on job embeddedness among new nurses from a theoretical point of view.

Based on the findings of previous studies, this study proposes the following hypotheses:

#### Hypothesis 1

The nursing work environment positively affects new nurses’ job embeddedness.

#### Hypothesis 2

Head nurse leadership mediates the effect of the nursing work environment on new nurses’ job embeddedness.

#### Hypothesis 3

Presenteeism mediates the effect of the nursing work environment on new nurses’ job embeddedness.

#### Hypothesis 4

Head nurse leadership and presenteeism sequentially mediate the effect of the nursing work environment on new nurses’ job embeddedness.

## Methods

### Study design and participants

An online self-reported cross-sectional multicentre study was conducted from November 2022 to January 2023; we collected data from many different individuals at a single point in time without any external influence [31]. This article was written in accordance with the Strengthening the Reporting of Observational Studies in Epidemiology (STROBE) guidelines [32].

Our target population was new nurses working in hospitals in Hunan Province. New nurses were operationally defined as nurses who had entered the workforce within the previous two years [33]. Therefore, registered nurses willing to participate who had obtained a nursing certificate and had been working in the clinic for ≤ 24 months were included in this study. Trainee nurses, refresher nurses, visiting scholars and those who could not continue participating for various reasons during the survey period were excluded.

### Data collection and bias

This study was conducted in hospitals in Hunan Province, China, where 29,700 new registered nurses began working between January 2020 and April 2022, representing an increase of 12% across the two-year period [34]. A two-stage convenience sampling method was adopted for the study. First, we selected 10 cities from different geographical regions of Hunan Province (3 in the central region, 2 in the northern region, 1 in the western region, 2 in the eastern region and 2 in the southern region). Second, we selected 3 tertiary hospitals (hospitals with more than 501 beds that provide high-level medical and health services, higher education and scientific research to several regions) and one secondary hospital (a 101–500-bed regional hospital providing comprehensive health services, teaching and research to multiple communities) from each city. As a result, 10 cities (Changsha, Hengyang, Shaoyang, Yiyang, Loudi, Chenzhou, Xiangtan, Zhuzhou, Yongzhou and Yueyang) and 33 hospitals (24 tertiary hospitals and 9 secondary hospitals) participated. The online questionnaire was produced through an online survey platform called WenJuanXing (https://www.wjx.cn/). We invited hospital administrators to display recruitment posters for our study on their bulletin boards and share them among their work groups; participants were provided with QR codes for self-registration, which they scanned to enter our WeChat group. The research team described the research content, data privacy and survey participation requirements of the WeChat group and then sent the survey link to each participant after providing informed consent.

We made the following efforts to address potential sources of bias. First, participants were selected from central, northern, southern, western, and eastern Hunan provinces to reduce regional selection bias. Second, we recruited 10 new nurses and conducted a presurvey to check for possible method bias. Therefore, we assumed that each account could be filled in only once to prevent duplication. Participants could submit the survey only after a period of more than 10 minutes to prevent fraud. Participants were required to complete all the questions before submitting to the survey to prevent missing data. Finally, to reduce nonresponse bias, we provided a monetary subsidy of 50 RMB for each participant. The initial response rate in the western region was low but increased substantially after we increased offline recruitment and expanded the subsidies.

### Sample size

We calculated the sample size necessary for this cross-sectional survey using the formula N = (Z_1−α/2_σ/δ)^2^, where α indicates the significance level, i.e., 0.05; Z_1−α/2_ is 1.96; σ indicates the standard deviation of the population; and δ indicates the allowable error. In a previous study, the mean score and standard deviation of nurses’ job embeddedness score were 22.87 ± 4.18 [35]; in this study, δ was 22.87 × 2%=0.4574. Therefore, the minimum sample size required was 321; however, considering a 20% potential dropout rate, the final sample size selected was 386. Of the 581 nurses registered for this study, 32 withdrew because they were no longer interested, and 64 did not meet the inclusion criteria. A total of 485 individuals completed the survey, for a response rate of 83.48%. After multivariate extreme value and outlier tests, 49 samples with obvious anomalies were deleted, and 436 were retained.

### Ethical considerations

This study was approved by the ethics review committee for behavioural medicine and nursing research of the Third Xiangya Hospital of Central South University (No. I 22,244, Changsha, Hunan, China). In addition, we obtained agreement and support from the leaders of all the targeted hospitals. We clarified the aim and content of the study to the participating nurses through WeChat groups, and WeChat’s data centre and WenJuanXing platform were used to assure that all the data would be used for research purposes only. All participants signed an electronic informed consent form and were informed that they could withdraw at any time without explanation or impact on their career.

### Variables and measures

In this study, the dependent variable was job embeddedness, while the predictive variables were the nursing work environment, head nurse leadership and presenteeism. We also included sex, age, hospital grade, weekly working hours, weekly number of night shifts, monthly salary and whether individuals worked independently (work with a teacher or work alone) as confounding variables in this study. These variables were selected according to a review study on job embeddedness-related variables [15].

The nursing work environment was measured by the Nursing Work Environment Scale developed by Shao Jing et al. [36], which comprises 26 items across 7 dimensions of career development (leadership and management, medical relationships, recognition atmosphere, professional autonomy, basic security and sufficient manpower). The scale adopts a Likert 6-point scoring method, with points 1 and 6 representing “strongly disagree” and “strongly agree”, respectively. The sum of the scores of each item reflects the status of the total scale or corresponding subscale. The higher the score is, the better the corresponding environmental characteristics will be. The Cronbach’s α coefficient of the scale was 0.946, and the split-half reliability was 0.894. In this study, the Cronbach’s α coefficient of the working environment scale was 0.982. Principal component analysis was conducted using the variance rotation method to extract 7 common factors from the study samples, accounting for 87.525% of the variance, and the factor loading range was 0.798–0.945.

The job embeddedness of the new nurses was evaluated by the Global Job Embeddedness Scale compiled by Crossley et al. [37] and revised by Mei Hua et al [38]. The scale comprises seven single-dimensional items and a 5-point Likert scale

was adopted: 5 means strongly agree, and 1 means strongly disagree. Items 4 and 6 are reverse-scored on a scale of 7 to 35, with a higher score indicating a higher level of job embeddedness. This scale has been widely used among nurses, with a Cronbach’s α ranging from 0.845–0.864 [35, 39]. In this study, the Cronbach’s α coefficient of the scale was 0.775, and the variance rotation method was used to extract a common factor from the sample in the principal component analysis to explain 76.414% of the variance. The loading range of the other factors ranged from 0.666 to 0.837.

We used the head nurse leadership scale developed by Huang Chunmei et al. [40] to evaluate head nurse leadership. This scale includes 6 dimensions: charisma, affinity, foresight, influence, decisiveness and power of control. Forty-four items were scored using a 5-point Likert scale (1 = never, 2 = occasionally, 3 = partially, 4 = often, 5 = always). The higher the total score is, the stronger the leadership of the head nurse. The Cronbach’s α of the scale was 0.988, the retest reliability of the questionnaire was 0.791, and the correlation coefficient between each item and the population was 0.597–0.854. In this study, the Cronbach’s α of the scale was 0.994, and 5 common factors were extracted from the principal component variance rotation analysis, with an explanatory variance of 89.446%. Among them, decisiveness and the power of control combined to constitute one factor. The factor loading range was 0.822–0.934.

The Stanford Presenteeism Scale was used to estimate the presenteeism of new nurses; this scale was developed at Stanford University in the U.S [25]. and translated into Chinese by Zhao Fang et al [41]. The scale comprises 6 items assessed using a 5-point Likert scale ranging from 1 to 5 points (completely disagree to completely agree). The higher the score is, the greater the degree of recessive absenteeism and the greater the productivity loss. The Cronbach’s α of each dimension of the scale ranged from 0.76 to 0.90. In this study, the Cronbach’s α of the scale was 0.919. The variance rotation method was used in the principal component analysis to extract a working factor from the study sample, explaining 92.035% of the variance. The factor loading range was 0.805–0.950.

### Quantitative and statistical analysis

We assigned binary data (sex, independent work, and hospital grade) values of 0 and 1. We arranged the hierarchical data (weekly working hours, weekly night shifts, and wage level) in order from 1 to 4 and treated them as continuous data in the subsequent statistical analyses.

Descriptive statistics were used for the sociodemographic characteristics and clinical performance levels of the participants. Count data, such as educational level, hospital grade, and whether the nurse worked independently, and ordinal data, such as weekly working hours, monthly income and weekly night shifts, were described as frequencies and constituent ratios. Quantitative data with absolute values of kurtosis and skewness less than 2, such as age, job embeddedness, the nursing work environment, head nurse leadership and presenteeism, were described as the mean and standard deviation [42]. Before statistical inference, a common method deviation test was performed with the Harman single-factor test. The results showed that the first factor, which was not rotated, explained only 32.93% of the total variance and did not account for 40% of the total variance. Path analyses were subsequently performed using PROCESS (version 3.5). According to the recommendations of Hayes [43], we examined the key research questions with sequential multiple mediation model analyses. Before model construction, biserial correlation and Pearson’s correlation coefficients were used to examine the correlations between all variables to discover correlation paths and possible confounding variables. Next, we used Model 6 to examine the sequential multiple mediating effects of head nurse leadership and presenteeism on the relationships between variables. To ensure the credibility of the results, we (1) standardized all the data, (2) used bias-corrected 5000 bootstrapped confidence intervals (CIs) to assess the statistical significance of the indirect mediation effects and (3) controlled for confounding factors.

## Results

### Participant characteristics and descriptive statistics

The mean age of the participants in this study was 23 (22,24), among whom 10.55% were male and 89.45% were female. The participants’ degrees were mainly junior (54.36%) or undergraduate (44.37%) at the college level, and 87.39% were individuals who worked independently. The participants mainly worked in tertiary hospitals (85.55%). A total of 71.33% of the participants worked more than 40 h per week, 69.95% earned less than US$735 per month, and they worked one (50.00%) or two (21.10%) night shifts per week. The job embeddedness score of the participants was 25.33 ± 4.51. The nursing work environment, head nurse leadership and presenteeism scores were 127.42 ± 22.45, 192.31 ± 29.35 and 15.84 ± 6.22, respectively. More detailed participant demographic and clinical characteristics are shown in Table 1.

**Table 1:** Demographics, nursing work environment, job embeddedness, head nurse leadership and presenteeism of the participants (*n* = 436)

Variables	N (%)	M(SD)	Min–Max
**Demographics (N/%)**			
Age, years		23 (1.79)	18–32
Gender			
Male	46 (10.55)	-	
Female	390 (89.45)	-	
Educational level			
Junior college	237 (54.36)	-	
Undergraduate	195 (44.73)	-	
Master degree or above	4 (0.92)	-	
Whether to work independently			
Yes	381 (87.39)	-	
No	55 (12.62)	-	
Hospital grade			
Tertiary	373 (85.55)	-	
Secondary	63 (14.45)	-	
Weekly working hours			
≤ 40	125 (28.67)	-	
> 40 and ≤ 50	279 (63.99)	-	
> 50	32 (7.34)	-	
Monthly income			
< 5000(approximately US$ 735)	305 (69.95)	-	
5000–8000(approximately US$ 735–1177)	125 (28.67)	-	
> 8000(approximately US$ 1177)	6 (1.38)	-	
Weekly night shifts			
0	61 (13.99)	-	
1	218 (50.00)	-	
2	92 (21.10)	-	
3 and above	65 (14.91)	-	
**Variable score (M/**SD**)**			
Nursing Work Environment	-	127.42 (22.45)	26–156
Job Embedding	-	25.33 (4.51)	11–35
Head Nurse Leadership	-	192.31 (29.35)	88–220
Presenteeism	-	15.84 (6.22)	6–30

Note: M = mean; SD = standard deviation

### Correlations among demographics, nursing work environment, head nurse leadership, presenteeism and job embeddedness

The correlation analysis showed that the nursing work environment (*r* = 0.731, *p* < 0.01) and head nurse leadership (*r* = 0.632, *p* < 0.01) were positively correlated with job embeddedness. Presenteeism (*r*=-0.349, *p* < 0.01), hospital grade (*r*=-0.112, *p* < 0.05), weekly working hours (*r*=-0.160, *p* < 0.05) and the number of night shifts per week (*r*=-0.100, *p* < 0.05) were negatively correlated with job embeddedness. The nursing work environment was positively correlated with head nurse leadership (*r* = 0.753, *p* < 0.01) and negatively correlated with presenteeism (*r*=-0.301, *p* < 0.01). There was a negative correlation between head nurse leadership and presenteeism (*r*=-0.335, *p* < 0.01). More detailed results regarding these variables are provided in Table 2.

**Table 2:** Correlations among demographic variables, nursing work environment, job embeddedness, head nurse leadership and presenteeism (*n* = 436)

Variables	JE	NWE	HNL	P	HG	WWH	WNS
**Job Embeddedness**	1						
**Nursing Work Environment**	0.731^**^						
**Head Nurse Leadership**	0.632^**^	0.753^**^					
**Presenteeism**	-0.349^**^	-0.301^**^	-0.335^**^				
**Hospital Grade**	0.112^*^	0.092	-0.026	-0.030			
**Weekly Working Hours**	-0.160^*^	-0.159^**^	-0.190^**^	0.062	0.042		
**Weekly Night Shifts**	-0.100^*^	-0.054	-0.095^*^	0.086	0.043	0.097^*^	1

Note: JE = job embeddedness; NWE = nursing work environment; HNL = head nurse leadership; P = presenteeism; HG = hospital grade; WWH = weekly working hours; WNS = weekly night shifts; ^*^p < 0.05; ^**^p < 0.01

### Sequential multiple mediation effects of head nurse leadership and presenteeism on the relationship between nursing work environment and job embeddedness

**Fig. 1:**
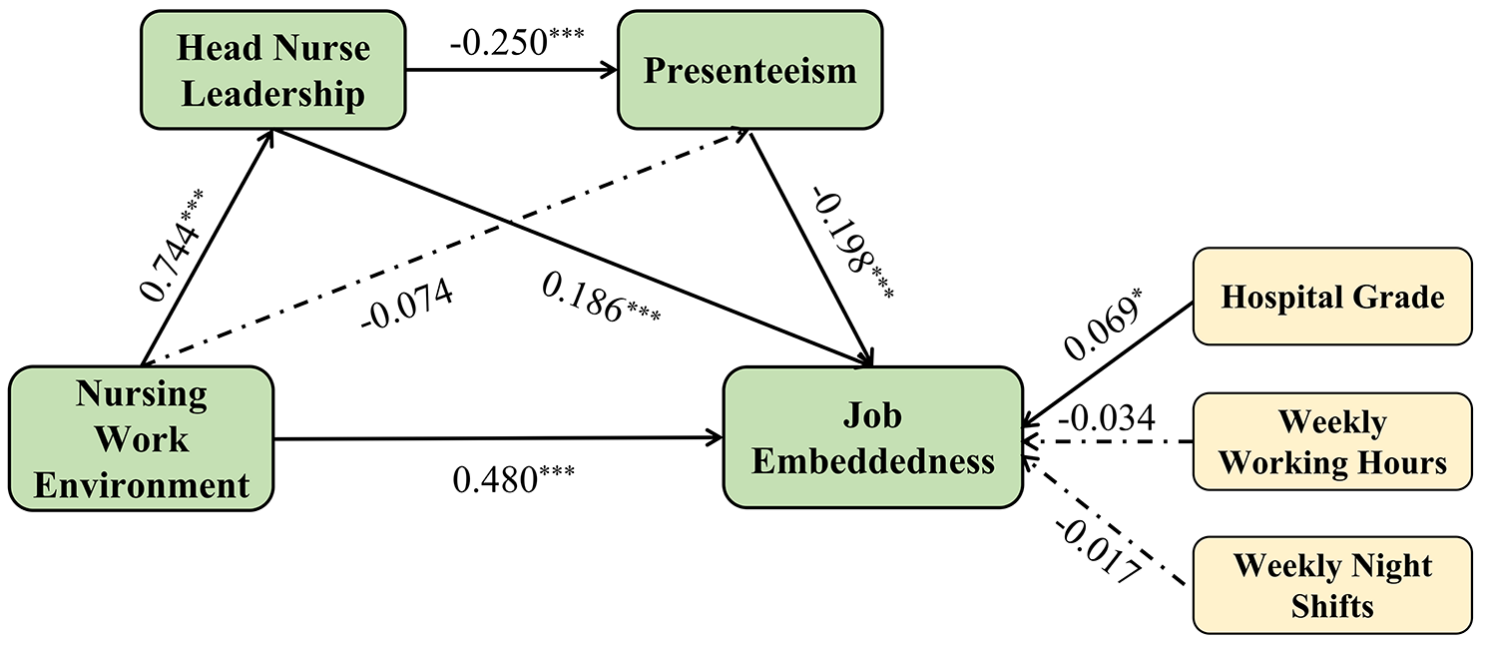
The sequential multiple mediating effects of head nurse leadership and presenteeism on the relationship between the nursing work environment and job embeddedness

Since hospital grade, weekly hours and weekly night shifts are associated with the job embeddedness of new nurses, we controlled for these factors when constructing the model to prevent confusion. Table 3 shows that the sequential multiple mediation model is well structured(*R*^*2*^ = 0.535, *F* = 82.160, *p* < 0.001). The complete model results are shown in Fig. 1. The nursing work environment directly affects the job embeddedness of new nurses (*β* = 0.480, *p* < 0.001). The nursing work environment also indirectly affects the job embeddedness of new nurses through the simple mediating effect of head nurse leadership *(β* = 0.139, *p* < 0.001) and the sequential mediating effect of head nurse leadership and presenteeism *(β* = 0.190, *p* < 0.001). The total effect of the nursing work environment on the job embeddedness of new nurses was se = 0.670, the direct effect was se = 0.480, accounting for 71.64%, and the indirect effect through the sequential mediating effect of nursing leadership and presenteeism was 0.190, accounting for 28.36%. More information is shown in Table 4.

**Table 3:** The sequential multiple mediating effects of head nurse leadership and presenteeism on the relationship between the nursing work environment and job embeddedness (*n* = 436)

Variables	Job Embeddedness (Y)				95% Confidence interval Conclusion	
	β	SE	t value	p	Boot LL	Boot UL
**Nursing Work Environment (X)**	0.480	0.051	9.481	0.000 ^***^	0.380	0.579
**Head Nurse Leadership (M**_**1**_ **)**	0.186	0.052	3.601	0.000 ^***^	0.085	0.288
**Presenteeism (M**_**2**_ **)**	-0.198	0.035	-5.684	0.000 ^***^	-0.266	-0.129
**Hospital Grade (C**_**1**_ **)**	0.069	0.033	2.075	0.039 ^*^	0.004	0.135
**Weekly Working Hours (C**_**2**_ **)**	-0.034	0.034	-1.009	0.314	-0.101	0.032
**Weekly Night Shifts (C**_**3**_ **)**	-0.017	0.033	-0.498	0.619	-0.082	0.049
***R***	0.731					
**Adjusted** ***R*** ^**2**^	0.535					
***F***	82.160^***^(*p* < 0.001)					

Note: M = mediator; C = covariate; SE = standard error; X = independent variable; Y = dependent variable; LL = lower limit; UL = upper limit; ^***^p < 0.001; ^*^p < 0.05

**Table 4:** Evaluation of the multiple sequential mediating effects of the nursing work environment on job embeddedness (*n* = 436)

Category	Hypothesized relationships	β	SE	95% Confidence interval Conclusion		Effect size
				Boot LL	Boot UL	
**Total effect**		0.670	0.035	0.600	0.739	100%
**Direct effect**	NWE– JE(H1)	0.480	0.051	0.380	0.579	71.64%
**Mediation effect**		0.190	0.055	0.073	0.280	28.36%
**Submediating effect**	NWE - HNL - JE(H2)	0.139	0.057	0.020	0.230	20.75%
	NWE - P - JE(H3)	0.015	0.014	-0.012	0.042	Unsupported
	NWE - HNL - P - JE(H4)	0.037	0.012	0.017	0.063	5.52%

Note: JE = job embeddedness; NWE = nursing work environment; HNL = head nurse leadership; P = presenteeism; SE = standard error; LL = lower limit; UL = upper limit

## Discussion

This study, which was based on COR theory, analysed the shaping mechanism of new nurses’ job embeddedness and proposed resource caravans and passageways for the nursing work environment, head nurse leadership and presenteeism, which are key to new nurses’ job embeddedness.

### Job embeddedness in new nurses

New nurses’ job embeddedness in this study was moderate and lower than that of senior nurses [44]. The longer the working experience of nurses is, the more connected they are with the hospital and the more resources and promotion opportunities they obtain; thus, experienced nurses exhibit deeper job embeddedness. In contrast, less experienced nurses use fewer resources and adapt less easily to heavy work tasks; thus, new nurses are less embedded in their work [45]. According to the COR theory, nurses who are poorly embedded at work cannot obtain more support resources through social relationships to improve key skill reserves and problem-solving abilities when facing work challenges, resulting in poor work performance [46]. Second, nurses with poor embeddedness cannot fully utilize their work resource reserves to effectively cope with potential losses caused by work pressure or work overload, resulting in mental health problems or abnormal organizational citizenship [47]. Most importantly, organizational fit in job embeddedness was strongly correlated with the retention of nurses from one to three years [48]. Overall, there is much room for improvement in new nurse job embeddedness. Furthermore, improving nurses’ job embeddedness levels to promote nurse retention is a necessary measure.

### Direct effect of the nursing work environment

For hypothesis 1, this study confirmed that the nursing work environment has a direct positive impact on the job embeddedness of new nurses, which is similar to previous research results on general nurses [45]. The contribution of the work environment to nurses’ job embeddedness is multifaceted. First, providing new nurses with learning opportunities will give them a sense of belonging, which will help them fit the organization, and this fit with the organization is also enhanced when new nurses experience that their values are consistent with those practised in the workplace [49]. Second, sufficient manpower reserves, high-quality medical teams and good medical-nursing cooperation models can promote mutual help, effective communication and full trust among nurses, thereby strengthening their connection with the organization and organizational personnel [13]. In the long run, the work environment is the basic resource of embeddedness since many other resources are rooted in this ecological environment. Therefore, building a good nursing work environment is particularly important for improving nurses’ job embeddedness.

### Simple mediating effect of head nurse leadership

For hypothesis 2, this study confirmed that head nurse leadership has a simple mediating effect on the relationship between the nursing work environment and new nurses’ job embeddedness. First, head nurses are also beneficiaries of resources. A supportive working environment can inspire individuals’ ownership, confidence and motivation, thereby improving their clinical leadership capabilities [23]. A high level of head nurse leadership can further promote job embeddedness [50]. Specifically, different leadership types have different mechanisms for job embedding. Servant leaders offer their employees a blend of resources, including social, positional, and organizational resources, which are essential for nurturing the job embeddedness of nurses [16]. Agile leaders use strategies such as group decision-making, problem solving, effective internal and external communication, and adaptation to uncertain environments to increase employees’ job embeddedness [44]. This process is consistent with the resource investment hypothesis. When new nurses obtain high-quality initial resources, that is, work environment resources, they benefit from downstream resources, that is, head nurse leadership [18]. The mutual promotion of resources is one way to shape new nurses’ job embeddedness.

### Simple mediating effect of presenteeism

For hypothesis 3, we found that presenteeism has no mediating role in the nursing work environment or job embeddedness but has a mediating role in head nurse leadership and job embeddedness. This is an unexpected discovery, but there are also clues that can be found in previous studies. First, the level of head nurse leadership is negatively related to presenteeism, as supportive leaders pay attention to nurses’ needs, interests and growth and provide them with emotional comfort and help, thereby reducing presenteeism [51]. Second, nurses’ presenteeism presents a risk to their health, increases job errors and burnout, and results in a loss of productivity and job embeddedness [52]. Combined with the primacy of the loss principle, when experiencing presenteeism, new nurses can recruit head nurse resources to buffer the loss of productivity resources and thereby achieve buffering embeddedness [53]. Notably, we found that when experiencing presenteeism, new nurses were more willing to recruit resources from head nurses than from the work environment. Resource accessibility may be a potential reason for this phenomenon. Although the nursing work environment contains higher-level leadership resources, the accessibility of these resources is poor. Head nurses are the leaders with the most daily contact and are the closest to new nurses; additionally, head nurses are the nearest upstream resource that is easier for nurses to access [22]. Requisitioning upstream resources to buffer the loss of a certain resource and its consequences is another way to shape the job embeddedness of new nurses.

### Sequential mediation of head nurse leadership and presenteeism

For hypothesis 4, the results revealed the sequential mediating effect of head nurse leadership and presenteeism on the relationship between the nursing work environment and new nurses’ job embeddedness. Additionally, previous research supports this result. A good nursing working environment enables nurses to communicate effectively with managers, and nurses have greater opinions of the leadership of head nurses [22]. Supportive leadership that gives nurses greater autonomy will effectively reduce presenteeism [51]. Less presenteeism among nurses means they are more committed to their work and show a greater level of job embeddedness [27]. Thus, improving the work environment is fundamental, and further improving head nurse leadership and preventing presenteeism can effectively help new nurses embed themselves in their work. COR theory also emphasizes the importance of initial resource accumulation. People who have been endowed with sufficient resources are less susceptible to resource losses and are more likely to obtain favourable resources, while people who lack resources may face increasing resource losses and fall into a spiral of resource loss [18]. Thus, initial resources, that is, the accumulation of the nursing work environment, are very important for shaping new nurses’ job embeddedness. The first step is to create a favourable work environment. Providing adequate human and material reserves, high-quality medical teams, a trustworthy organizational culture, a caring atmosphere and sufficient development opportunities are the basis for shaping the embeddedness of new nurses [47]. The second step is to improve the leadership of head nurses. Changes in leadership styles, such as humility, service and agility, can adapt to the needs of different new nurses or to different stages of the same nurse to flexibly adjust the embeddedness of new nurses [16, 44]. The third step is to reduce presenteeism. Managers need to re-examine the “irreplaceability” of a certain employee, pay attention to the physical and mental health of new nurses, and provide timely help to reduce presenteeism and make them comfortable with their work [54].

### Implications for nursing management

Numerous practical implications for nursing management can be derived from the results of this study. First, it is necessary to care about new nurses’ job embeddedness to retain new nurses. Second, managers can shape new nurses’ job embeddedness by providing work environment resources, leadership resources, and individual productivity resources. Specifically, they should be equipped with sufficient organizational manpower; build reasonable medical-nursing cooperation models; provide new nurses with sufficient learning and development opportunities; cultivate different head nurse leadership styles according to the needs of new nurses, such as service leadership style, agile leadership style, and supportive leadership style; pay attention to the physical and mental health of new nurses; and provide timely help and support.

## Conclusions

This study reported that the job embeddedness of new nurses needs to be improved. These results suggest that preserving adequate resources for new nurses, such as work environment resources, head nurse leadership resources, and individual productivity resources, is an effective way to shape their job embeddedness. In addition, when a certain resource is insufficient, fully considering the principles of investment and buffering between resources and providing reciprocal, alternative, or buffer resources in a timely manner are necessary to improve new nurses’job embeddedness.

### Limitations and future directions

A rigorous scientific design was implemented in this study, but several limitations remain. First, the study recruited participants from hospitals in only the Hunan Province of China due to limited funding and time, which may have resulted in regional bias. Second, this was a cross-sectional study; thus, causal inferences between variables could not be derived. Third, although quality control was in place before the questionnaires were distributed, some invalid questionnaires were still generated due to limitations in the online questionnaire software, which may have affected the authenticity of the findings. Based on this study, it is necessary to further consider the influence of different environmental factors, organizational management factors and personal factors (including physiological and psychological) on the job embeddedness of new nurses and to enrich the understanding of the prefactors and theoretical connotations of job embeddedness. In addition, future research should consider additional resources and verify the effectiveness of COR theory in explaining job embeddedness.

### Electronic supplementary material

Below is the link to the electronic supplementary material.

Supplementary Material 1

Supplementary Material 2

## Data Availability

All data generated or analysed during this study are included in this published article [and its supplementary information files].

## Abbreviations

JE: Job embeddedness
NEW: Nursing work environment
HNL: Head nurse leadership
P: Presenteeism
