# Supplementary material for: The role of the nursing work environment, head nurse leadership and presenteeism in job embeddedness among new nurses: a cross-sectional multicentre study
Source: BMC Nurs. 2024 Mar 5;23:159. doi: 10.1186/s12912-024-01823-1 (PMC10913553; doi:10.1186/s12912-024-01823-1)
Supplement: Supplementary file 2 — Supplementary Material 2 [file 12912_2024_1823_MOESM2_ESM.docx]

**Table S1.** **Demographics Questionnaire about participants**

| Age |  | Gender |  |
| --- | --- | --- | --- |
| Educational level | □ Junior college □ Undergraduate  □ Master degree or above | | |
| Hospital grade | □ Secondary-C □ Secondary-B □ Secondary-A  □ Tertiary-C □ Tertiary-B □ Tertiary-A | | |
| Monthly income | □ <5000rmb □ 5000~8000rmb □ ˃8000rmb | | |
| Whether to work independently | □ Yes □ No | | |
| Weekly working hours | □ ≤40h‎ □ 40～50h □ ˃50h | | |
| Weekly night shifts | □ 0 □ 1  □ 2 □ 3 and above | | |

**Table S2. The Nursing Work Environment Scale**

| Please read each of the following statements and decide how well it re­flects your thoughts and feelings about other people in general. There is no right or wrong answer. Using the response scale, from 1 to 6, circle the degree to which you agree or disagree with each statement directly on the booklet. Please answer all questions. | |
| --- | --- |
| Items | Strongly Strongly  disagree agree |
| 1. The hospital supports my on-the-job education. | 1 2 3 4 5 6 |
| 1. The hospital can provide me with the reference books and Internet resources I need | 1 2 3 4 5 6 |
| 3. The hospital can provide me with personalized development space | 1 2 3 4 5 6 |
| 4. The hospital can provide me with career planning guidance and encourage me to set learning goals and career planning | 1 2 3 4 5 6 |
| 5. The hospital can provide me with a perfect pre-job training and teaching plan | 1 2 3 4 5 6 |
| 6. When it comes to decisions, the leader listens to my advice | 1 2 3 4 5 6 |
| 7. When I made a mistake, my leader encouraged me to learn from it rather than condemn it | 1 2 3 4 5 6 |
| 8. The leader is good at listening and can give corresponding answers to my concerns | 1 2 3 4 5 6 |
| 9. Leaders will praise my ideas when appropriate | 1 2 3 4 5 6 |
| 10. The doctors respected my observations and judgment | 1 2 3 4 5 6 |
| 11. The doctors respected my professionalism | 1 2 3 4 5 6 |
| 12. The doctors recognized my contribution to the patient's recovery | 1 2 3 4 5 6 |
| 13. I have a good relationship with the doctor | 1 2 3 4 5 6 |
| 14. I am recognized in my work by patients and their families | 1 2 3 4 5 6 |
| 15. I am recognized in my work by other medical professionals | 1 2 3 4 5 6 |
| 16. I feel that my work is worthwhile | 1 2 3 4 5 6 |
| 17. I am able to make independent decisions regarding patient care and work | 1 2 3 4 5 6 |
| 18. I believe it is important to accept and respect the attitudes and feelings of others. | 1 2 3 4 5 6 |
| 19.I had the opportunity to participate in quality improvement projects | 1 2 3 4 5 6 |
| 20.When I encounter a problem, I first rely on my own ability to solve it | 1 2 3 4 5 6 |
| 21.I am satisfied with the benefits I have received | 1 2 3 4 5 6 |
| 22.I am satisfied with the time off I have received | 1 2 3 4 5 6 |
| 23.I am satisfied with the salary I received | 1 2 3 4 5 6 |
| 24.I have enough time to communicate with my patients | 1 2 3 4 5 6 |
| 25.I had ample time and opportunity to discuss patient care with other nurses | 1 2 3 4 5 6 |
| 26.The allocation of nursing human resources can meet the clinical needs | 1 2 3 4 5 6 |

**Table S3. The Global Job Embedding Scale**

| Below you will find a set of statements followed by numbers from 1 to 5. Please read each statement carefully. After reading the statement, decide how well it describes you. If you strongly agree with a statement, circle 5. If, however, you strongly disagree with a statement, then circle 1. There are no right or wrong answers. Answer as honestly as possible. Please read and answer all items. | |
| --- | --- |
| Items | Strongly Strongly  disagree agree |
| 1. I feel attached to my work | 1 2 3 4 5 |
| 2. I had a hard time making the decision to leave the organization | 1 2 3 4 5 |
| 3. I care too much about this job to leave | 1 2 3 4 5 |
| 4. It's easy for me to leave my present workplace | 1 2 3 4 5 |
| 5. I really can't leave my present job lightly | 1 2 3 4 5 |
| 6. I'm bored with my work unit | 1 2 3 4 5 |
| 7. I feel very connected to my work | 1 2 3 4 5 |

Note: Items 4 and 6 were scored in reverse

**Table S4. The Head Nurse Leadership Scale**

| The following questions will understand your evaluation of the leadership of head nurses in the current department. There are a set of statements followed by numbers from 1 to 5. Please read each statement carefully. If you think the head nurse never did this, circle 1, and if you think the head nurse always did, circle 5. After reading the statement, decide how well it describes you. There are no right or wrong answers. Answer as honestly as possible. Please read and answer all items. | |
| --- | --- |
| Items | Never Always |
| 1. Make team members clear about their responsibilities | 1 2 3 4 5 |
| 2. Rational use and management of materials, equipment and consumables | 1 2 3 4 5 |
| 3.To implement the post competency training for nurses | 1 2 3 4 5 |
| 4. Clearly express the work objectives and requirements of the department | 1 2 3 4 5 |
| 5. Ensure the time for nurses to complete nursing tasks | 1 2 3 4 5 |
| 6.Effectively strive for the support of various departments of nursing work | 1 2 3 4 5 |
| 7.Pay attention to the cooperation and communication between departments, and integrate the relationship with relevant departments, doctors, and patients | 1 2 3 4 5 |
| 8.Nurses have fair opportunities for learning, training, promotion and evaluation | 1 2 3 4 5 |
| 9.Flexible scheduling according to nursing workload | 1 2 3 4 5 |
| 10.Scheduling can take into account the reasonable wishes of nurses | 1 2 3 4 5 |
| 11.Performance appraisal is objective, fair and transparent | 1 2 3 4 5 |
| 12.Have the ability to identify problems, can dialectically look at the existing problems | 1 2 3 4 5 |
| 13. Clearly grasp the quality standards and requirements of the department | 1 2 3 4 5 |
| 14. Can lead the team to complete conscientiously according to quality standards and requirements | 1 2 3 4 5 |
| 15. Department goals were set according to the cost and benefit of care | 1 2 3 4 5 |
| 16. Develop specific steps to achieve the goals of care | 1 2 3 4 5 |
| 17. Make good use of human and material resources to achieve nursing goals | 1 2 3 4 5 |
| 18. Can effectively resolve all kinds of contradictions and quell disputes | 1 2 3 4 5 |
| 19.Able to make quick decisions and deal with emergencies | 1 2 3 4 5 |
| 20.Can effectively organize the rescue of critical patients | 1 2 3 4 5 |
| 21.Appropriate ways can be used to motivate nurses | 1 2 3 4 5 |
| 22.The degree of motivation matched the performance of the nurses | 1 2 3 4 5 |
| 23.Encourage and recognize nurses when they successfully complete their work | 1 2 3 4 5 |
| 24.Taken into account nurses' abilities and interests when delegating | 1 2 3 4 5 |
| 25.Trust and empower nurses and give guidance | 1 2 3 4 5 |
| 26.Regularly evaluate the achievement of the goals of the empowerment program | 1 2 3 4 5 |
| 27.Encourage nurses to participate in the management of the department | 1 2 3 4 5 |
| 28.Able to maintain the quality of care in the department at a high level | 1 2 3 4 5 |
| 29.Be sincere, honest, fair and just | 1 2 3 4 5 |
| 30.Respect nurses' beliefs, privacy and personal interests | 1 2 3 4 5 |
| 31.Emotional stability and good self-control | 1 2 3 4 5 |
| 32.Recognize individual differences and treate nurses' problems with "empathy" | 1 2 3 4 5 |
| 33.Have the communication style of empathy | 1 2 3 4 5 |
| 34.Create an atmosphere of communication and problem solving among nurses | 1 2 3 4 5 |
| 35.Care about the personal life of nurses and try to help solve difficulties | 1 2 3 4 5 |
| 36.Have certain ability of nursing research | 1 2 3 4 5 |
| 37.Pay attention to the cultivation of clinical and scientific research innovation ability of nurses | 1 2 3 4 5 |
| 38.Have a certain clinical work innovation ability | 1 2 3 4 5 |
| 39.Have the ability and behavior to develop specialized nursing | 1 2 3 4 5 |
| 40.Motivate and encourage nurses to implement new professional standards | 1 2 3 4 5 |
| 41.Understand the trend of nursing development in your specialty | 1 2 3 4 5 |
| 42.Love nursing post and is full of confidence in nursing career | 1 2 3 4 5 |
| 43.Hace the patient-centered service concept | 1 2 3 4 5 |
| 44.Have passion, can stimulate the work enthusiasm of nurses | 1 2 3 4 5 |

**Table S5. The Stanford Presenteeism Scale**

| Below you will find a set of statements followed by numbers from 1 to 5. Please read each statement carefully. After reading the statement, decide how well it describes you. If you strongly agree with a statement, circle 5. If, however, you strongly disagree with a statement, then circle 1. There are no right or wrong answers. Answer as honestly as possible. Please read and answer all items. | |
| --- | --- |
| Items | Strongly Strongly  disagree agree |
| 1. Over the past month, my work stress has become even more difficult to manage because of my health problems | 1 2 3 4 5 |
| 2.For the past month, health problems have prevented me from completing difficult tasks at work | 1 2 3 4 5 |
| 3. For the past month, health problems have prevented me from enjoying my work | 1 2 3 4 5 |
| 4. For the past month, I have found it impossible to carry out certain work tasks because of health issues | 1 2 3 4 5 |
| 5. For the past month, despite my health problems, I have been able to concentrate on my work | 1 2 3 4 5 |
| 6. For the past month, despite my health problems, I still feel energetic enough to get all my work done | 1 2 3 4 5 |
